# Age and sex-related outcomes in cardiovascular magnetic resonance versus computed tomography-guided transcatheter aortic valve replacement: a secondary analysis of a randomized clinical trial

**DOI:** 10.1016/j.jocmr.2025.101882

**Published:** 2025-03-13

**Authors:** Ivan Lechner, Fritz Oberhollenzer, Christina Tiller, Magdalena Holzknecht, Alex Kaser, Ronald K. Binder, Can Gollmann-Tepeköylü, Gert Klug, Agnes Mayr, Axel Bauer, Bernhard Metzler, Martin Reindl, Sebastian J. Reinstadler

**Affiliations:** a University Clinic of Internal Medicine III, Cardiology and Angiology, Medical University of Innsbruck, Innsbruck, Austria; b Department of Cardiology and Intensive Care, University Teaching Hospital Klinikum Wels-Grieskirchen, Wels, Austria; c University Clinic of Cardiac Surgery, Medical University of Innsbruck, Innsbruck, Austria; d Department of Internal Medicine, County Hospital Bruck an der Mur, Bruck an der Mur, Austria; e University Clinic of Radiology, Medical University of Innsbruck, Innsbruck, Austria

**Keywords:** Transcatheter aortic valve replacement, Cardiac magnetic resonance imaging, Cardiac computed tomography, Age

## Abstract

**Background:**

Transcatheter aortic valve replacement (TAVR) is the preferred treatment for older patients with severe aortic stenosis with outcomes influenced by age and sex. Computed tomography (CT) is the reference imaging modality for TAVR planning, while cardiovascular magnetic resonance (CMR) is an emerging alternative for this indication. The aim of this study was to evaluate the impact of age and sex on implantation success in patients undergoing CT- or CMR-guided TAVR.

**Methods:**

This was a secondary analysis of the randomized TAVR-CMR trial comparing TAVR planning by CT or CMR (NCT03831087). Patients were categorized according to the median age (82 years) and sex. Implantation success, defined using the Valve Academic Research Consortium-2 definition (absence of procedural mortality, correct positioning of a single prosthetic valve, and proper prosthetic valve performance), was compared at hospital discharge between age groups and sex for each imaging strategy. All-cause mortality at 6 months was compared between imaging strategies across age groups and sex.

**Results:**

A total of 267 patients (median age 82 [IQR 80–85] years, 50% (133/267) female) underwent TAVR at two heart centers in Austria between September 2017 and December 2022. Implantation success did not differ significantly between imaging strategies across age and sex subgroups. For patients ≤82 years, success rates were 92.1% (58/63) (CT) vs. 94.7% (72/76) (CMR) (p = 0.524), and for those >82 years, 89.4% (59/66) (CT) vs. 91.9% (57/62) (CMR) (p = 0.622). Among female patients, success rates were 84.7% (50/59) (CT) vs. 93.2% (69/74) (CMR) (p = 0.113), and among male patients, 95.7% (67/70) (CT) vs. 93.8% (60/64) (CMR) (p = 0.610). All-cause mortality at 6 months did not differ significantly between imaging strategies across age and sex subgroups. Mortality rates for patients ≤82 and >82 years were 4.8% (3/63) vs. 5.3% (4/76) (p = 0.839) and 9.1% (6/66) vs. 12.9% (8/62) (p = 0.490) for CT and CMR, respectively. Similarly, female and male patients had comparable mortality rates (10.2% (6/59) vs. 8.1% (6/74), p = 0.680; 4.3% (3/70) vs. 9.4% (6/64), p = 0.240).

**Conclusion:**

In this secondary analysis of the TAVR-CMR trial, CMR-guided TAVR was associated with similar outcomes compared with CT-guided TAVR irrespective of age and sex.

## Graphical abstract

**Figure:**
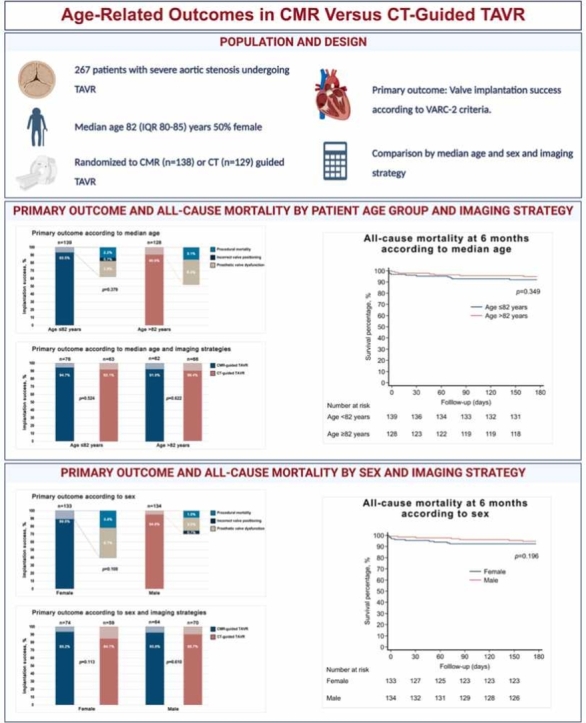
*CMR* cardiac magnetic resonance, *CT c*omputed tomography, *TAVR t*ranscatheter aortic valve replacement, *VARC* valve academic research consortium, *IQR* interquartile range. Created with Biorender.

## 1. Introduction

Transcatheter aortic valve replacement (TAVR) is rapidly evolving [1], with indications extending beyond the elderly to younger patients [2]. Indeed, TAVR has emerged as a less invasive alternative to surgical aortic valve replacement across the full spectrum of surgical risk [3], [4], [5], [6], [7], [8], [9], [10]. Pre-procedural imaging is a key component of TAVR planning to ensure accurate assessment of the access route and valve landing zone. Computed tomography (CT) has become the standard imaging modality for this purpose [11]. However, other imaging modalities, such as cardiac magnetic resonance (CMR) may offer a safe and radiation-free alternative [12], [13].

The recent TAVR-CMR (*Cardiac Magnetic Resonance Imaging Versus Computed Tomography to Guide Transcatheter Aortic Valve Replacement*) trial demonstrated that CMR-guided TAVR was non-inferior to CT-guided TAVR in terms of device implantation success, adverse clinical outcomes at discharge and 6-month mortality [14]. However, the different pathophysiological profiles of younger versus older patients, including differences in aortic valve calcification, vascular characteristics, and comorbidities [15], warrant an in-depth investigation of whether imaging strategies differentially affect TAVR outcomes across age groups. Furthermore, as TAVR expands into younger populations [2], understanding the role of radiation- and iodinated contrast free imaging modalities such as CMR is relevant to long-term patient safety and procedural success [12], [16].

In addition to age, the natural differences in pre-procedural imaging characteristics between female and male patients undergoing TAVR may have potential implications for clinical outcomes [17], [18]. Female patients generally have smaller landing zone and access-site parameters [18], [19], [20], [21]. These sex differences are critical in establishing a new guiding strategy for TAVR to avoid inaccuracies in pre-procedural imaging and prevent potential sex-related procedural risks. However, there is a lack of evidence for possible sex-based differences in imaging parameters and clinical outcomes according to the imaging strategy used to guide TAVR.

The aim of this analysis was therefore to investigate the potential influence of age and sex on the outcomes of patients who were randomized to either CMR- or CT-guided TAVR planning.

## 2. Methods

### 2.1. Study design

TAVR-CMR was an investigator-initiated, prospective, randomized, open-label, non-inferiority trial conducted at two Austrian heart centers. The detailed study design and results of the trial have been previously published [14]. In brief, potential TAVR candidates who met the eligibility criteria and consented to participate were randomly assigned in a 1:1 ratio to undergo either a predefined TAVR-CMR protocol or a standard contrast-enhanced TAVR-CT protocol to assess the anatomical characteristics of the aortic annulus and access route.

The main inclusion criteria were as follows: (I) severe aortic stenosis diagnosed according to the European Society of Cardiology/European Association for Cardio-Thoracic Surgery guidelines (aortic valve area ≤1.0 cm² or aortic valve index ≤0.6 cm²/m²) and (II) typical symptoms of severe aortic stenosis.

Main exclusion criteria were (I) presence of absolute contraindications to CMR or CT; (II) contraindications to TAVR; (III) reduced life expectancy <1 year; (IV) severe renal insufficiency requiring renal replacement therapy; and (V) severe hepatic insufficiency (Child-Pugh class B or C).

The primary outcome was defined according to Valve Academic Research Consortium (VARC)−2 criteria [22] as device implantation success at discharge including following components: (I) absence of procedural mortality; (II) correct positioning of a single prosthetic heart valve into the proper anatomical location; and (III) proper intended performance of the prosthetic heart valve (mean aortic valve gradient <20 mmHg and no valve regurgitation >mild).

Secondary outcomes included the individual components of the primary outcome: all-cause mortality, stroke or transient ischemic attack (TIA), life-threatening bleeding, acute kidney injury (stage 2 or 3 including kidney replacement therapy), coronary artery obstruction requiring intervention, major vascular complications, valve-related dysfunction requiring repeat procedure, and need for the implantation of a permanent pacemaker at discharge. In addition, VARC-3 device success criteria were assessed, defined as a composite of technical success, freedom from mortality, freedom from surgery or intervention related to the device or to a major vascular or access-related or cardiac structural complication, and intended valve performance [23]. Finally, all-cause mortality was assessed from the electronic medical record by data retrieval from the federal institution Statistics Austria at 6 months after discharge.

The trial was conducted in accordance with the ethical principles of the 1975 Declaration of Helsinki and approved by the Ethics Committee of the Medical University of Innsbruck (study number: AN2017–0056 371/4.11 474/AM 1). The trial is registered on ClinicalTrials.gov (NCT03831087).

In this secondary analysis of the TAVR-CMR trial, we compared CMR-guided TAVR with CT-guided TAVR between younger and older patients, defined by median age, and between female and male patients.

### 2.2. Imaging procedures

For TAVR-CMR, CMR examinations were performed using a 1.5 Tesla Magnetom AVANTO-scanner (Siemens Healthineers, Erlangen, Germany). The detailed imaging protocol has been published previously [14], [24]. Briefly, the applied CMR protocol included a non-contrast-enhanced, electrocardiographically (ECG)-triggered, navigator-gated, free breathing three-dimensional (3D) whole-heart acquisition using a steady-state free precession sequence covering the range between left ventricular outflow tract to the ascending aorta. For planning of this sequence, a cubic 3D volume was placed at the level of the bulbus aortae as defined at axial, coronal and parasagittal localizers. Additionally, a coronal 3D fast low-angle shot (FLASH) gadolinium (Gd)-magnetic resonance angiography (MRA) sequence before and after the intravenous administration of 0.2 mmol/kg of Gd-DO3A-butriol (Gadovist, Bayer Healthcare, Berlin, Germany) at 2 mL/s was performed. The acquisition volume for the MRA sequence extended from supra-aortal branches to the femoral artery.

CT scans were performed on a 128-slice dual-source CT (128 mm × 0.6 mm detector collimation, 0.28 s gantry rotation time) with high-pitch factor (3.2; Somatom Definition Flash, Siemens Healthineers, Forchheim, Germany). Prospective electrocardiographically synchronization was applied and a bolus of 70 to 110 mL of nonionic iodine contrast agent with a concentration of 370 mg/ml (Iopromide, Ultravist 370, Bayer Healthcare, Berlin, Germany) was injected, using an automatic injector at a flow rate of 5 mL/s. Contrast agent volume was calculated by scan time and body weight. The scan region ranged from the supraaortic branches to the femoral artery.

The aortic annulus (area, perimeter and diameters) was measured manually on both, properly reconstructed and rotated 3D whole-heart magnetic resonance angiography and CT images [25]. Complete standardized peripheral TAVR planning including aortic and iliac artery sizing was performed on FLASH contrast-enhanced MRA and on CT images.

### 2.3. Statistical analysis

In this secondary analysis, the intention-to-treat population was analyzed. Statistical analysis was performed with SPSS Statistics 29.0.1 (IBM, Armonk, New York), and MedCalc Version 22.0.22 (MedCalc Software Ltd, Ostend, Belgium). Patients were grouped by median age into those ≤ median and > median and by sex. Distribution of data was tested using the Shapiro–Wilk test. Categorical variables are depicted as frequencies with corresponding percentages. Continuous variables are presented as mean ± standard deviation (SD) or median with interquartile range (IQR), according to their distribution. Differences between groups were tested with Student’s t-test, Mann–Whitney U-test, or Chi-square test as indicated.

For analyses of 6-month mortality, time-to-event was defined as time from the TAVR procedure to the outcome date; Kaplan–Meier curves were conducted, and log-rank comparisons were used to evaluate between group differences.

A two-tailed p-value of <0.05 was considered as statistically significant.

## 3. Results

### 3.1. Baseline patient imaging characteristics

In TAVR-CMR, 380 potential TAVR candidates were randomized to CMR-guided (191 patients) or CT-guided (189 patients) TAVR planning, of which 267 patients eventually underwent TAVR. Of the 267 patients, 138 were randomized in the CMR-guided group and 129 patients in the CT-guided group.

Median age of the study cohort was 82 (IQR 80–85) years, 50% (133/267) were female.

Of the 380 potential TAVR candidates randomized in TAVR-CMR, there was no significant age and sex-related difference between patients who underwent TAVR (267 patients) and those who did not (113 patients) for patients ≤82 and >82 years (66% (139/212) vs. 76% (128/168), p = 0.124), as well as female and male patients (69% (133/194) vs. 72% (134/186), p = 0.457).

Protocol deviations were comparable between female and male patients. A total of 10 patients (3.8% (10/267)) experienced a crossover from CMR to CT or vice versa, including 5 female (1.9% (5/267)) and 5 male (1.9% (5/267)) patients. Additionally, 9 patients (3.4% (9/267)) in the TAVR-CMR group required an additional CT scan for TAVR planning, comprising 5 female (1.9% (5/267)) and 4 male (1.5% (4/267)) patients.

Patient demographics and imaging characteristics according to median age (≤82 and >82) and sex are summarized in Table 1 and Table 2, respectively. Patient demographics and imaging data were not significantly different between patients ≤82 and >82 years of age, except for a lower body-mass index (25 [IQR: 22–27] vs. 26 [IQR: 24–29], p<0.001), less frequent history of previous coronary artery bypass graft (2% (3/128) vs. 10% (14/139), p = 0.010) and a smaller aortic valve area (0.6 [IQR: 0.5–0.7] vs. 0.7 [IQR: 0.5–0.8] cm^2^, p = 0.005) observed in the older patient group. Similarly, no significant differences have been observed in female and male patients, except for a lower rate of previous PCI (27% (36/133) vs. 46% (61/134), p<0.001), coronary artery bypass graft (3% (4/133) vs. 10% (13/134), p = 0.025) and chronic pulmonary disease (9% (12/133) vs. 19% (26/134), p = 0.015) in female patients. Furthermore, female patients showed a significant smaller aortic valve area (0.6 [IQR: 0.5–0.7] cm^2^ vs. 0.7 [IQR: 0.6–0.9] cm^2^, p<0.001) and significantly higher left ventricular (LV) ejection fraction (58 [52–65] % vs. 55 [50–62] %, p = 0.011). In addition, female patients demonstrated significantly smaller landing zone parameters, vessel dimensions, ostial heights, and femoral access route vessel dimensions (all p<0.001, Table 2).

**Table 1:** Baseline characteristics according to median age and sex.

	Total population (n = 267)	Age ≤82 (n = 139)	Age >82 (n = 128)	p-value	Female (n = 133)	Male (n = 134)	p-value
Randomization allocation				0.308			0.198
TAVR-CMR group	138 (52)	76 (55)	62 (48)		74 (52)	64 (48)	
TAVR-CT group	129 (48)	63 (45)	66 (52)		59 (44)	70 (56)	
Age (years)	82 [80–85]	80 [78–82]	85 [84–87]	**<0.001**	83 [81–85]	82 [79–86]	0.422
Female sex	133 (50)	65 (47)	68 (53)	0.299			
Body-mass index (kg/m²)	25 [23–28]	26 [24–29]	25 [22–27]	**<0.001**	25 [22–28]	26 [24–29]	0.058
STS Score	4 [4-5]	4 [4-5]	4 [4-5]	0.897	4 [4-5]	4 [4-5]	0.848
Previous myocardial infarction	38 (14)	23 (17)	15 (12)	0.259	14 (11)	24 (18)	0.084
Previous CABG	17 (6)	14 (10)	3 (2)	**0.010**	4 (3)	13 (10)	**0.025**
Previous PCI	77 (29)	46 (33)	31 (24)	0.110	36 (27)	61 (46)	**<0.001**
Previous stroke/TIA	33 (12)	18 (13)	15 (12)	0.760	13 (10)	20 (15)	0.201
Atrial fibrillation	96 (36)	50 (36)	46 (36)	0.995	45 (34)	51 (38)	0.472
PAD	15 (7)	10 (7)	5 (4)	0.244	5 (4)	10 (8)	0.189
COPD	38 (14)	18 (13)	20 (16)	0.532	12 (9)	26 (19)	**0.015**
Chronic liver disease	14 (5)	9 (7)	5 (4)	0.593	6 (5)	8 (6)	0.593
Chronic kidney disease §	108 (40)	55 (40)	53 (41)	0.760	75 (56)	68 (51)	0.355
Estimated glomerular filtration rate (mL/min/1.73m^2^)	58 [44–70]	60 [44–74]	56 [44–61]	0.234	55 [42–74]	58 [47–72]	0.069
Aortic valve morphology				0.469			0.414
Tricuspid	261 (98)	135 (97)	126 (98)		131 (99)	130 (97)	
Bicuspid	6 (2)	4 (3)	2 (2)		2 (2)	4 (3)	
Aortic valve area (cm²)	0.6 [0.5–0.8]	0.7 [0.5–0.8]	0.6 [0.5–0.7]	**0.005**	0.6 [0.5–0.7]	0.7 [0.6–0.9]	**<0.001**
Mean gradient (mmHg)	40 [30–46]	40 [30–46]	40 [30–46]	0.876	41 [31–49]	42 [33–51]	0.102
Moderate or severe aortic regurgitation	63 (24)	32 (23)	31 (24)	0.818	33 (25)	30 (22)	0.641
Left ventricular ejection fraction (%)	57 [49–64]	57 [47–62]	58 [51–64]	0.197	58 [52–65]	55 [50–62]	**0.011**
Stroke volume index (ml/m²)	30 [25–39]	31 [24–39]	29 [25–38]	0.825	29 [24–35]	32 [25–41]	**0.026**

Data are n (%), and median (IQR). *TAVR* transcatheter aortic valve replacement, *CMR c*ardiac magnetic resonance, *STS* Society of Thoracic Surgeons, *CABG* coronary artery bypass grafting, *PCI* percutaneous coronary intervention, *TIA* transient ischemic attack, *PAD* peripheral arterial disease, *COPD* chronic obstructive pulmonary disease. § Defined as Kidney Disease Improving Global Outcomes (KDIGO) scores 3a and 3b

**Table 2:** Imaging and transcatheter aortic valve replacement data according to median age and sex.

	Total population (n = 267)	Age ≤82 (n = 139)	Age >82 (n = 128)	p-value	Female (n = 133)	Male (n = 134)	p-value
Atrial fibrillation during scanning	67 (25)	36 (26)	31 (24)	0.592	26 (20)	41 (31)	0.074
Annulus area (mm²)	441 [376–500]	445 [382–500]	438 [364–503]	0.408	387 [347–441]	487 [440–552]	**<0.001**
Minimum annulus diameter (mm)	21 [19–23]	21 [19–23]	21 [19–23]	0.857	20 [18–21]	22 [21–24]	**<0.001**
Maximum annulus diameter (mm)	26 [24–28]	26 [25–29]	26 [24–28]	0.270	25 [23–27]	28 [26–29]	**<0.001**
Annulus perimeter (mm)	77 [71–82]	77 [71–81]	76 [71–82]	0.455	72 [68–77]	81 [77–86]	**<0.001**
Sinus of Valsalva diameter (mm)	32 [29–35]	32 [29–35]	31 [28–34]	0.428	30 [28–32]	34 [31–36]	**<0.001**
Sinotubular junction diameter (mm)	27 [25–30]	27 [25–30]	27 [24–29]	0.152	25 [23–28]	28 [26–31]	**<0.001**
Ascending aorta diameter (mm)	34 [32–37]	35 [32–37]	34 [32–37]	0.684	33 [30–35]	35 [33–38]	**<0.001**
Moderate or severe calcification of aortic valve leaflets and left ventricular outflow tract	241 (90)	127 (94)	114 (89)	0.089	124 (93)	117 (87)	0.111
Ostial height RCA (mm)	15 [±3]	14 [±3]	15 [±3]	0.871	14 [±3]	15 [±3]	**<0.001**
Ostial height LM (mm)	14 [±2]	14 [±2]	14 [±2]	0.062	13 [±2]	14 [±2]	**0.007**
Minimum diameter of peripheral vessels							
A. right femoral (mm)	7.5 [6.5–8.5]	7.4 [6.3–8.5]	7.8 [6.5–8.6]	0.472	7.0 [6.0–7.9]	8.0 [7.0–9.0]	**<0.001**
A. right iliac (mm)	8.6 [±2.0]	8.4 [±2.1]	8.8 [±1.9]	0.290	8.0 [±2]	9.0 [±2]	**<0.001**
A. left femoral·(mm)	7.3 [6.5–8.7]	7.2 [6.5–8.4]	7.8 [6.3–8.8]	0.361	7.0 [6.0–8.0]	8.2 [7.0–9.0]	**<0.001**
A. left iliac (mm)	8.0 [1.1–9.5]	8.0 [7.0–9.3]	8.1 [7.5–9.8]	0.252	8.0 [7.0–9.0]	8.6 [7.0–10.0]	**<0.001**
Moderate or severe calcification of the right access site	107 (40)	56 (40)	51 (40)	0.712	50 (38)	57 (43)	0.250
Moderate or severe calcification of the left access site	120 (45)	59 (42)	61 (48)	0.627	57 (43)	63 (47)	0.270
Access routes for TAVR				0.160			0.109
Trans-femoral	254 (95)	131 (94)	123 (96)		120 (98)	124 (94)	
Trans-apical	4 (1)	1 (1)	3 (2)		2 (2)	2 (2)	
Trans-axillary	7 (3)	5 (4)	2 (2)		0 (0)	7 (5)	
Trans-aortic	2 (1)	2 (1)	0 (0)		1 (<1)	1 (1)	
TAVR types				0.777			0.196
Balloon-expandable	232 (87)	120 (86)	112 (87.5)		112 (84)	120 (90)	
Self-expanding	35 (13)	19 (14)	16 (12.5)		21 (16)	14 (10)	

Data are n (%), and median (IQR). *TAVR* transcatheter aortic valve replacement, *CMR* cardiac magnetic resonance, *RCA* right coronary artery, *LM* left main coronary artery, *fem*, femoralis

Patient demographics and imaging characteristics by median age and by their respective imaging strategy are summarized in Table 3, Table 4, respectively.

**Table 3:** Baseline characteristics according to median age and imaging strategies.

	Total population (n = 267)	Age ≤82 (n = 139)			Age >82 (n = 128)		
		TAVR-CMR group n = 76	TAVR-CT group n = 63	p-value	TAVR-CMR group n = 62	TAVR-CT group n = 66	p-value
Age (years)	82 [80–85]	80 [78–81]	80 [77–82]	0.679	85 [84–87]	85 [84–88]	0.532
Female sex	133 (50)	37 (49)	28 (44)	0.618	37 (60)	31 (47)	0.150
Body-mass index (kg/m²)	25 [23–28]	26 [24–29]	26 [24–30]	0.968	25 [23–27]	24 [22–27]	0.486
STS Score	4 [4,5]	4 [4,5]	4 [4,5]	0.571	4 [4,5]	4 [4,5]	0.488
Previous myocardial infarction	38 (14)	13 (17)	10 (16)	0.846	7 (11)	8 (12)	0.884
Previous CABG	17 (6)	7 (9)	7 (11)	0.711	1 (2)	2 (3)	0.596
Previous PCI	77 (29)	21 (28)	25 (40)	0.133	18 (29)	13 (20)	0.218
Previous stroke/TIA	33 (12)	10 (13)	8 (13)	0.936	8 (13)	7 (11)	0.686
Atrial fibrillation	96 (36)	27 (36)	23 (37)	0.904	23 (37)	23 (35)	0.791
COPD	38 (14)	11 (15)	7 (11)	0.557	10 (16)	10 (15)	0.879
Chronic kidney disease §	108 (40)	30 (40)	25 (40)	0.980	29 (47)	24 (36)	0.232
Estimated glomerular filtration rate (ml/min/1.73 m^2^)	58 [44–70]	60 [43–73]	59 [46–77]	0.744	53 [39–62]	60 [48–61]	0.098
Aortic valve morphology				0.226			0.964
Tricuspid	261 (98)	75 (99)	60 (95)		61 (98)	65 (98.5)	
Bicuspid	6 (2)	1 (1)	3 (5)		1 (2)	1 (1.5)	
Aortic valve area (cm²)	0.6 [0.5–0.8]	0.7 [0.5–0.9]	0.7 [0.6–0.8]	0.549	0.6 [0.5–0.7]	0.6 [0.5–0.8]	0.413
Mean gradient (mmHg)	40 [30–46]	40 [29–48]	40 [31–46]	0.943	41 [31–46]	40 [29–47]	0.983
Moderate or severe aortic regurgitation	63 (24)	17 (22)	15 (24)	0.841	18 (29)	13 (20)	0.218
Left ventricular ejection fraction (%)	57 [49–64]	55 [48–61]	58 [46–62]	0.407	56 [48–64]	59 [53–65]	0.146
Stroke volume index (ml/m²)	30 [25–39]	31 [25–38]	32 [24–40]	0.892	28 [24–36]	31 [25–40]	0.129

Data are n (%), and median (IQR). *TAVR* transcatheter aortic valve replacement, *CMR c*ardiac magnetic resonance, *STS* society of thoracic surgeons, *CABG* coronary artery bypass grafting, *PCI* percutaneous coronary intervention, *TIA* transient ischemic attack, *COPD* chronic obstructive pulmonary disease. § Defined as Kidney Disease Improving Global Outcomes (KDIGO) scores 3a and 3b

**Table 4:** Imaging and transcatheter aortic valve replacement data according to median age and imaging strategies.

	Total population (n = 267)	Age ≤82 (n = 139)			Age >82 (n = 128)		
		TAVR-CMR group n = 76	TAVR-CT group n = 63	p-value	TAVR-CMR group n = 62	TAVR-CT group n = 66	p-value
Atrial fibrillation during scanning	67 (25)	21 (28)	15 (24)	0.564	15 (24)	16 (24)	0.995
Annulus area (mm²)	441 [376–500]	451 [381–508]	441 [382–494]	0.738	431 [351–506]	442 [370–501]	0.180
Minimum annulus diameter (mm)	21 [19–23]	21 [19–22]	21 [19–23]	0.978	21 [18–22]	21 [20–23]	**0.013**
Maximum annulus diameter (mm)	26 [24–28]	27 [25–29]	26 [24–28]	0.219	26 [24–28]	26 [24–27]	0.820
Annulus perimeter (mm)	77 [71–82]	77 [71–82]	77 [71–81]	0.849	76 [68–82]	77 [72–83]	0.276
Sinus of Valsalva diameter (mm)	32 [29–35]	33 [30–36]	31 [28–31]	**0.001**	32 [29–35]	31 [28–34]	0.079
Sinotubular junction diameter (mm)	27 [25–30]	26 [24–29]	28 [26–31]	**<0.001**	26 [24–28]	27 [24–30]	0.142
Ascending aorta diameter (mm)	34 [32–37]	35 [32–36]	35 [32–38]	0.629	35 [31–36]	34 [32–37]	0.816
Ostial height RCA (mm)	15 [±3]	15 [±3]	14 [±3]	0.104	15 [±3]	15 [±3]	0.167
Ostial height LM (mm)	14 [±2]	14 [±3]	13 [±2]	0.288	14 [±2]	14 [±2]	0.507
Minimum diameter of peripheral vessels							
A. right femoral (mm)	7.5 [6.5–8.5]	7.4 [6.5–9.0]	7.3 [6.3–8.2]	0.456	8.0 [6.0–9.0]	7.7 [6.7–8.4]	0.791
A. right iliac (mm)	8.6 [±2.0]	8.6 [±2.1]	8.1 [±2.0]	0.202	8.9 [±2.1]	8.7 [±1.6]	0.059
A. left femoral·(mm)	7.3 [6.5–8.7]	7.0 [7.0–9.0]	7.2 [6.1–8.0]	0.094	8.0 [6.0–9.0]	7.8 [6.8–8.8]	0.807
A. left iliac (mm)	8.0 [1.1–9.5]	8.7 [7.0–10]	8.0 [6.9–8.8]	**0.013**	8.9 [8.0–10.0]	8.0 [7.5–9.2]	0.071
Access routes for TAVR				0.297			0.276
Trans-femoral	254 (95)	73 (96)	58 (92)		58 (94)	65 (98)	
Trans-apical	4 (1)	1 (1.3)	0 (0)		3 (5)	(0)	
Trans-axillary	7 (3)	1 (1.3)	4 (6)		1 (1)	1 (2)	
Trans-aortic	2 (1)	1 (1.3)	1 (2)		0 (0)	0 (0)	
TAVR types				0.236			0.141
Balloon-expandable	232 (87)	68 (89.5)	52 (82.5)		57 (92)	55 (83)	
Self-expanding	35 (13)	8 (10.5)	11 (17.5)		5 (8)	11 (17)	

Data are n (%), and median (interquartile range). *TAVR* transcatheter aortic valve replacement, *CMR c*ardiac magnetic resonance, *RCA* right coronary artery, *LM* left main coronary artery, *fem* femoralis

Patient demographics were not significantly different between imaging strategies for patients ≤82 and >82 years of age. Imaging data were also comparable between groups, with the exception of a smaller sinus of Valsalva diameter (31 [IQR: 28–31] vs. 33 [IQR: 30–36] mm, p = 0.001) and a smaller left iliac artery diameter (8.0 [IQR: 6.9–8. 8] vs. 8.7 [IQR: 7.0–10.0] mm, p = 0.013) and larger sinotubular junction diameter (28 [IQR: 26–31] vs. 26 [IQR: 24–29] mm, p<0.001) in the CT-guided TAVR group in patients ≤82 years of age. Additionally, a larger minimum annulus diameter was observed in patients >82 years of age undergoing CT-guided TAVR (21 [IQR: 20–23] vs. 21 [IQR: 18–22] mm, p = 0.013). No significant relation of age with either imaging modality (CMR group vs. CT group 82 [80–85] vs. 83 [80–85] years, p = 0.350) has been observed.

Patient demographics and imaging characteristics by sex and by their respective imaging strategy are summarized in Table 5, Table 6, respectively. Patient demographics were not significantly different between imaging strategies for female and male patients. Imaging data were also comparable between groups, with the exception of a smaller Sinus of Valsalva diameter in the CT-guided group compared with the CMR-guided group among female (29 [IQR: 27–32] vs. 30 [IQR: 28–32] mm, p = 0.014) and male patients (32 [IQR: 30–34] vs. 36 [IQR: 33–37] mm, p<0.001). Additionally, left iliac artery diameter was smaller in the CT-guided group compared with the CMR-guided group in male patients (8.0 [IQR: 7.3–9.5] vs. 9.0 [IQR: 8.0–11.0] mm, p=0.006). Similarly, the right iliac artery diameter was smaller in the CT-guided group compared with the CMR-guided group in male patients (8.6 [±1.9] vs. 9.5 [±2.1] mm, p = 0.018). Conversely, the sinotubular junction diameter was larger in the CT-guided group compared with the CMR-guided group among female (26 [IQR: 24–28] vs. 25 [IQR: 23–27] mm, p = 0.018) and male patients (30 [IQR: 27–32] vs. 27 [IQR: 26–30] mm, p = 0.028).

**Table 5:** Baseline characteristics according to sex and imaging strategies.

	Total population (n = 267)	Female (n = 133)			Male (n = 134)		
		TAVR-CMR group n = 74	TAVR-CT group n = 59	p-value	TAVR-CMR group n = 64	TAVR-CT group n = 70	p-value
Age (years)	82 [80–85]	83 [81–85]	83 [80–85]	0.937	82 [78–84]	83 [79–87]	0.203
Female sex	133 (50)	-	-		0 (0)	0 (0)	
Body-mass index (kg/m²)	25 [23–28]	25 [22–28]	25 [23–30]	0.248	27 [25–29]	25 [23–28]	0.020
STS Score	4 [4-5]	4 [4-5]	4 [4-5]	0.882	4 [4-5]	4 [4-5]	0.950
Previous myocardial infarction	38 (14)	9 (12)	5 (9)	0.491	11 (17)	13 (19)	0.835
Previous CABG	17 (6)	2 (3)	2 (3)	0.818	6 (9)	7 (10)	0.903
Previous PCI	77 (29)	17 (23)	9 (15)	0.265	22 (34)	29 (41)	0.401
Previous stroke/TIA	33 (12)	7 (10)	6 (10)	0.891	11 (17)	9 (13)	0.482
Atrial fibrillation	96 (36)	24 (32)	21 (36)	0.702	26 (40)	25 (36)	0.559
COPD	38 (14)	7 (10)	5 (9)	0.844	14 (22)	12 (17)	0.489
Chronic kidney disease §	108 (40)	42 (57)	33 (56)	0.924	35 (55)	37 (53)	0.383
Estimated glomerular filtration rate (ml/min/1.73 m^2^)	58 [44–70]	56 [40–65]	56 [46–60]	0.937	56 [43–73]	60 [50–79]	0.131
Aortic valve morphology							
Tricuspid	261 (98)	73 (99)	58 (98)	0.872	63 (98)	67 (96)	0.355
Bicuspid	6 (2)	1 (1)	1 (1)	0.872	1 (2)	3 (4)	0.355
Aortic valve area (cm²)	0.6 [0.5–0.8]	0.5 [0.5–0.7]	0.6 [0.4–0.8]	0.129	0.7 [0.6–0.9]	0.7 [0.6–0.8]	0.053
Mean gradient (mmHg)	40 [30–46]	42 [33–51]	41 [28–45]	0.102	35 [27–46]	40 [32–46]	0.050
Moderate or severe aortic regurgitation	63 (24)	20 (27)	13 (22)	0.508	15 (23)	15 (21)	0.781
Left ventricular ejection fraction (%)	57 [49–64]	57 [51–64]	59 [53–65]	0.227	55 [40–60]	56 [47–63]	0.166
Stroke volume index (ml/m²)	30 [25–39]	28 [22–35]	30 [25–35]	0.202	32 [25–40]	33 [25–41]	0.848

Data are n (%), and median (IQR). *TAVR* transcatheter aortic valve replacement, *CMR c*ardiac magnetic resonance, *STS* society of thoracic surgeons, *CABG* coronary artery bypass grafting, *PCI* percutaneous coronary intervention, *TIA* transient ischemic attack, *COPD* chronic obstructive pulmonary disease. § Defined as Kidney Disease Improving Global Outcomes (KDIGO) scores 3a and 3b

**Table 6:** Imaging and transcatheter aortic valve replacement data according to sex and imaging strategies.

	Total population (n = 267)	Female (n = 133)			Male (n = 134)		
		TAVR-CMR group n = 74	TAVR-CT group n = 59	p-value	TAVR-CMR group n = 64	TAVR-CT group n = 70	p-value
Atrial fibrillation during scanning	67 (25)	16 (22)	10 (17)	0.519	20 (31)	21 (30)	0.875
Annulus area (mm²)	441 [376–500]	386 [336–441]	394.0 [356–442]	0.433	506 [450–545]	478 [438–543]	0.334
Minimum annulus diameter (mm)	21 [19–23]	19 [18–21]	20 [19–21]	0.174	22 [21–23]	22 [21–24]	0.202
Maximum annulus diameter (mm)	26 [24–28]	25 [24–27]	25 [23–26]	0.313	28 [26–31]	27 [26–29]	0.085
Annulus perimeter (mm)	77 [71–82]	71 [68–77]	73 [70–78]	0.280	82 [77–87]	80 [77–85]	0.144
Sinus of Valsalva diameter (mm)	32 [29–35]	30 [28–32]	29 [27–32]	**0.014**	36 [33–37]	32 [30–34]	**<0.001**
Sinotubular junction diameter (mm)	27 [25–30]	25 [23–27]	26 [24–28]	**0.018**	27 [26–30]	30 [27–32]	**0.028**
Ascending aorta diameter (mm)	34 [32–37]	34 [29–35]	33 [30–36]	0.605	35 [33–37]	35 [32–38]	0.901
Ostial height RCA (mm)	15 [±3]	12.8 [14.0–16.0]	13.0 [11.0–15.0]	0.091	16 [±3]	15 [±3]	0.283
Ostial height LM (mm)	14 [±2]	13.2 [12.0–15.0]	12.8 [11.8–14.3]	0.186	15 [±2]	14 [±2]	0.003
Minimum diameter of peripheral vessels							
A. right femoral (mm)	7.5 [6.5–8.5]	7.0 [6.0–8.0]	7.1 [6.4–7.9]	0.610	8.3 [7.0–9.0]	8.0 [6.8–8.9]	0.213
A. right iliac (mm)	8.6 [±2.0]	8.0 [7.0–9.0]	8.1 [7.0–9.0]	0.951	9.5 [±2.1]	8.6 [±1.9]	**0.018**
A. left femoral·(mm)	7.3 [6.5–8.7]	7.0 [6.0–8.0]	6.8 [6.0–7.7]	0.393	8.3 [7.0–9.7]	8.2 [7.1–9.0]	0.120
A. left iliac (mm)	8.0 [1.1–9.5]	8.0 [7.0–9.0]	7.8 [6.9–8.5]	0.084	9.0 [8.0–11.0]	8.0 [7.3–9.5]	**0.006**
Access routes for TAVR				0.077			0.351
Trans-femoral	254 (95)	72 (97)	58 (98)		59 (92)	65 (93)	
Trans-apical	4 (1)	2 (3)	-		2 (3)	-	
Trans-axillary	7 (3)	-	-		2 (3)	5 (7)	
Trans-aortic	2 (1)	-	1 (2)		1 (2)	-	
TAVR types				**0.025**			0.698
Balloon-expandable	232 (87)	67 (91)	45 (76)		58 (91)	62 (89)	
Self-expanding	35 (13)	7 (10)	14 (24)		6 (9)	8 (11)	

Data are n (%), and median (IQR). *TAVR* transcatheter aortic valve replacement, *CMR c*ardiac magnetic resonance, *RCA* right coronary artery, *LM* left main coronary artery, *fem* femoralis

Anatomical and hemodynamic parameters of the aortic valve are provided in Supplementary Table S1.

### 3.2. Primary outcome

The primary outcome of device implantation success was available for all patients who underwent TAVR and was similar in patients ≤82 and >82 years and between imaging strategies, as well as by sex and imaging strategy.

**Fig. 1:**
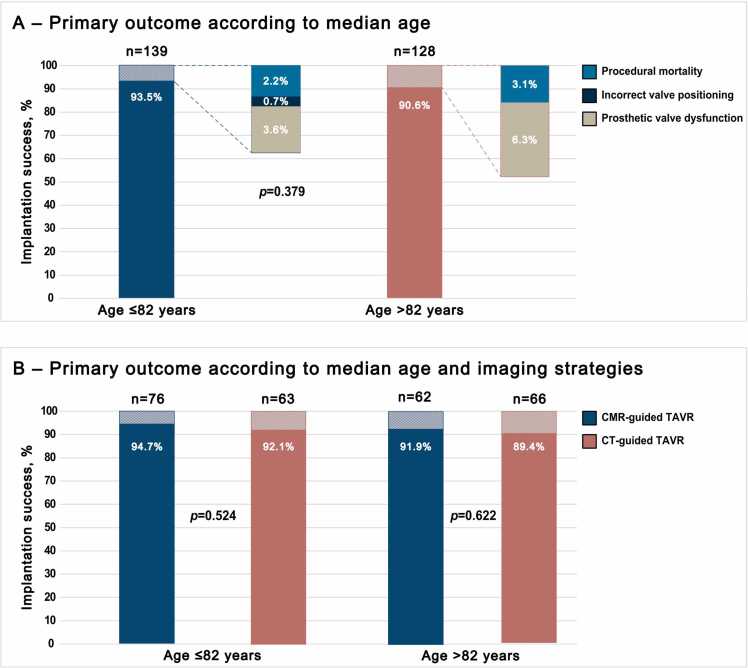
Primary outcome by patient age group and imaging strategy. *CMR* cardiovascular magnetic resonance*, CT* computed tomography*, TAVR* transcatheter aortic valve replacement

In patients ≤82 years of age, device implantation success was achieved in 130 of 139 patients (93.5% (130/139)) and in 116 of 128 patients (90.6% (116/128)) in patients >82 years of age (p = 0.379). The primary outcome according to age groups and its components are summarized in Table 7 and Fig. 1A.

**Table 7:** Clinical outcomes according to median age and sex.

	Total population (n = 267)	Age ≤82 (n = 139)	Age >82 (n = 128)	p-value	Female (n = 133)	Male (n = 134)	p-value
Primary outcome							
Device implantation success §	246 (92.1)	130 (93.5)	116 (90.6)	0.379	119 (89.5)	127 (94.8)	0.108
Procedural mortality	7 (2.6)	3 (2.2)	4 (3.1)	0.621	5 (3.8)	2 (5.2)	0.246
Incorrect valve positioning	1 (0.4)	1 (0.7)	0 (0.0)	0.336	1 (0.8)	1 (1)	0.996
Absence of intended performance	13 (4.9)	5 (3.6)	8 (6.3)	0.314	9 (6.8)	4 (3.0)	0.151
Secondary outcomes at discharge							
All-cause mortality	8 (3.0)	3 (2.2)	5 (3.9)	0.403	6 (4.5)	2 (1.5)	0.148
Stroke/TIA	8 (3.0)	5 (3.6)	3 (2.3)	0.548	5 (3.8)	3 (2.2)	0.466
Life-threatening bleeding	8 (3.0)	3 (2.2)	5 (3.9)	0.403	5 (3.8)	3 (2.2)	0.466
Acute kidney injury	52 (19.5)	25 (18.0)	27 (21)	0.522	21 (15.8)	31 (23.1)	0.130
Acute kidney injury based on retention parameters	49 (18.4)	22 (15.8)	27 (21.1)	0.267	19 (14.3)	30 (22.4)	0.087
Need for dialysis	3 (1.1)	3 (2.2)	0 (0.0)	0.095	2 (1.5)	1 (0.7)	0.557
Coronary artery obstruction requiring intervention	0 (0)	0 (0)	0 (0)	-	0 (0)	0 (0)	-
Major vascular complications	8 (3.0)	3 (2.2)	5 (3.9)	0.403	5 (3.8)	3 (2.2)	0.616
Valve-related dysfunction requiring repeat procedure	3 (1.1)	2 (1.4)	1 (0.8)	0.611	1 (0.8)	2 (1.5)	0.566
Permanent pacemaker	20 (7.5)	10 (7.2)	10 (7.8)	0.848	9 (6.8)	11 (8.2)	0.654
All-cause mortality at 6 months	21 (7.9)	7 (5.0)	14 (11.0)	0.074	12 (9.0)	9 (6.7)	0.484

Data are n (%), and median (IQR). *TAVR* transcatheter aortic valve replacement, *CMR* cardiovascular magnetic resonance, *TIA* transient ischemic attack. § absence of procedural mortality, correct positioning of a single prosthetic heart valve into the proper anatomical location, and proper intended performance of the prosthetic heart valve (mean aortic valve gradient < 20 mmHg and no valve regurgitation > mild) at discharge. ¶ valve regurgitation >mild, mean valve gradient >20 mmHg at discharge

The primary outcome and its components by age group and imaging strategy are summarized in Table 8. In patients ≤82 years, device implantation success was achieved in 72 of 76 patients (94.7%) in the CMR group and in 58 of 63 patients (92.1%) in the CT group (p = 0.524). In patients >82 years, device implantation success was achieved in 57 of the 62 CMR-guided TAVR group (91.9%) and in 59 of 66 CT-guided TAVR group (89.4%) (p = 0.622) (Fig. 1B).

**Table 8:** Clinical outcomes according to median age and imaging strategies.

	Total population (n = 267)	Age ≤82 (n = 139)			Age >82 (n = 128)		
		TAVR-CMR group n = 76	TAVR-CT group n = 63	p-value	TAVR-CMR group n = 62	TAVR-CT group n = 66	p-value
Primary outcome							
Device implantation success §	246 (92.1)	72 (94.7)	58 (92.1)	0.524	57 (91.9)	59 (89.4)	0.622
Procedural mortality	7 (2.6)	2 (2.6)	1 (1.6)	0.673	2 (3.2)	2 (3.0)	0.949
Incorrect valve positioning	1 (0.4)	0 (0)	1 (1.6)	0.270	0 (0)	0 (0)	-
Absence of intended performance	13 (4.9)	2 (2.7)	3 (4.7)	0.502	3 (4.8)	5 (7.6)	0.523
Secondary outcomes at discharge							
All-cause mortality	8 (3.0)	2 (2.6)	1 (1.6)	0.673	3 (4.8)	2 (3.0)	0.598
Stroke/TIA	8 (3.0)	1 (1.3)	4 (6.3)	0.113	0 (0)	3 (4.5)	0.089
Life-threatening bleeding	8 (3.0)	2 (2.6)	1 (1.6)	0.673	2 (3.2)	3 (4.5)	0.700
Acute kidney injury	52 (19.5)	15 (19.7)	10 (15.9)	0.555	10 (16.1)	17 (25.8)	0.182
Acute kidney injury based on retention parameters	49 (18.4)	14 (18.4)	8 (12.7)	0.357	10 (16.1)	17 (25.8)	0.182
Need for dialysis	3 (1.1)	1 (1.3)	2 (3.2)	0.453	0 (0)	0 (0)	-
Coronary artery obstruction requiring intervention	0 (0)	0 (0)	0 (0)	-	0 (0)	0 (0)	-
Major vascular complications	8 (3.0)	2 (2.6)	1 (1.6)	0.673	2 (3.2)	3 (4.5)	0.700
Valve-related dysfunction requiring repeat procedure	3 (1.1)	0 (0)	2 (3.2)	0.118	1 (1.6)	0 (0)	0.300
Permanent pacemaker	20 (7.5)	3 (3.9)	7 (11.1)	0.104	2 (3.2)	8 (12.1)	0.061
All-cause mortality at 6 months	21 (7.9)	4 (5.3)	3 (4.8)	0.839	8 (12.9)	6 (9.1)	0.490

Data are n (%), and median (interquartile range). *TAVR* transcatheter aortic valve replacement, *CMR* cardiovascular magnetic resonance, *TIA* transient ischemic attack. § absence of procedural mortality, correct positioning of a single prosthetic heart valve into the proper anatomical location, and proper intended performance of the prosthetic heart valve (mean aortic valve gradient <20 mmHg and no valve regurgitation > mild) at discharge. ¶ valve regurgitation >mild, mean valve gradient >20 mmHg at discharge

No significant relation between age and clinical outcomes (primary outcome of implantation success present vs. absent 82 [80–85] vs. 83 [79–86] years, p = 0.588) has been observed.

**Fig. 2:**
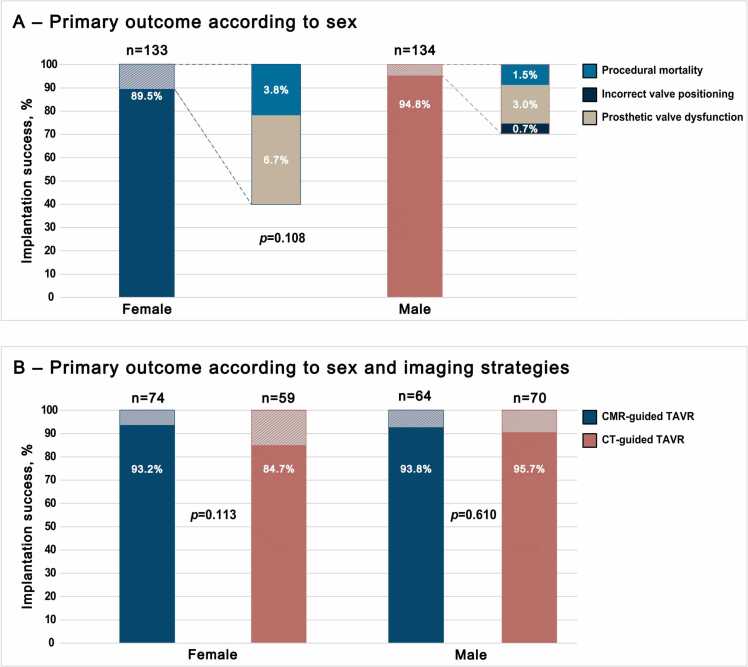
Primary outcome by patient sex and imaging strategy. *CMR* cardiovascular magnetic resonance*, CT computed tomography, TAVR* transcatheter aortic valve replacement

In female patients, device implantation success was achieved in 119 of 133 patients (89.5%), and in male patients in 127 of 134 patients (94.8%) (p = 0.108). The primary outcome according to sex and its components are summarized in Table 7 and Fig. 2A. The primary outcome and its components by sex and imaging strategy are summarized in Table 9. Among female patients, device implantation success was achieved in 69 of 74 patients (93.2%) in the CMR group and in 50 of 59 patients (84.7%) in the CT group (p = 0.113). Among male patients, device implantation success was achieved in 60 of 64 patients (93.8%) in the CMR group and in 67 of 70 patients (95.7%) in the CT group (p = 0.610) (Fig. 2B).

**Table 9:** Clinical outcomes according to sex and imaging strategies.

	Total population (n = 267)	Female (n = 133)			Male (n = 134)		
		TAVR-CMR group n = 74	TAVR-CT group n = 59	p-value	TAVR-CMR group n = 64	TAVR-CT group n = 70	p-value
Primary outcome							
Device implantation success §	246 (92.1)	69 (93.2)	50 (84.7)	0.113	60 (93.8)	67 (95.7)	0.610
Procedural mortality	7 (2.6)	2 (2.7)	3 (5.1)	0.473	2 (3.1)	0 (0.0)	0.136
Incorrect valve positioning	1 (0.4)	0 (0.0)	1 (1.7)	0.261	0 (0.0)	1 (1.4)	0.337
Absence of intended performance	13 (4.9)	3 (4.1)	6 (10.2)	0.163	2 (3.1)	2 (2.9)	0.927
Secondary outcomes at discharge							
All-cause mortality	8 (3.0)	3 (4.1)	3 (5.1)	0.776	2 (3.1)	0 (0)	0.136
Stroke/TIA	8 (3.0)	1 (1.4)	4 (6.8)	0.102	0 (0.0)	3 (4.3)	0.094
Life-threatening bleeding	8 (3.0)	2 (2.7)	3 (5.1)	0.473	2 (3.1)	1 (1.4)	0.507
Acute kidney injury	52 (19.5)	11 (14.9)	10 (16.9)	0.743	14 (21.9)	17 (24.3)	0.741
Acute kidney injury based on retention parameters	49 (18.4)	11 (14.9)	8 (13.6)	0.831	13 (20.3)	17 (24.3)	0.582
Need for dialysis	3 (1.1)	0 (0.0)	2 (3.4)	0.111	1 (1.6)	0 (0.0)	0.296
Coronary artery obstruction requiring intervention	0 (0)	0 (0.0)	0 (0.0)	-	0 (0.0)	0 (0.0)	-
Major vascular complications	8 (3.0)	2 (2.7)	3 (5.1)	0.601	2 (3.1)	1 (1.4)	0.849
Valve-related dysfunction requiring repeat procedure	3 (1.1)	1 (1.4)	0 (0.0)	0.370	0 (0.0)	2 (2.9)	0.173
Permanent pacemaker	20 (7.5)	3 (4.1)	6 (10.2)	0.163	2 (3.1)	9 (12.9)	0.040
All-cause mortality at 6 months	21 (7.9)	6 (8.1)	6 (10.2)	0.680	6 (9.4)	3 (4.3)	0.240

Data are n (%), and median (interquartile range). *TAVR* transcatheter aortic valve replacement, *CMR* cardiovascular magnetic resonance, *TIA* transient ischemic attack. § absence of procedural mortality, correct positioning of a single prosthetic heart valve into the proper anatomical location, and proper intended performance of the prosthetic heart valve (mean aortic valve gradient <20 mmHg and no valve regurgitation >mild) at discharge. ¶ valve regurgitation >mild, mean valve gradient >20 mmHg at discharge

### 3.3. Secondary outcomes

**Fig. 3:**
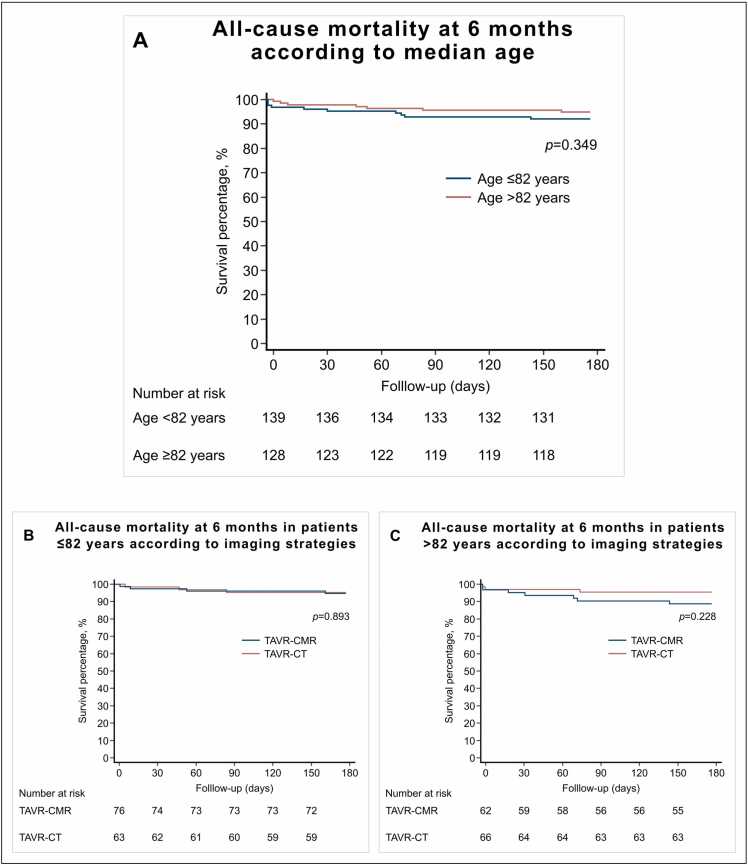
Cumulative incidence of all-cause mortality between patient age groups and imaging strategy. *CMR* cardiovascular magnetic resonance*, CT* computed tomography*, TAVR* transcatheter aortic valve replacement. *p*-value for log-rank.

**Fig. 4:**
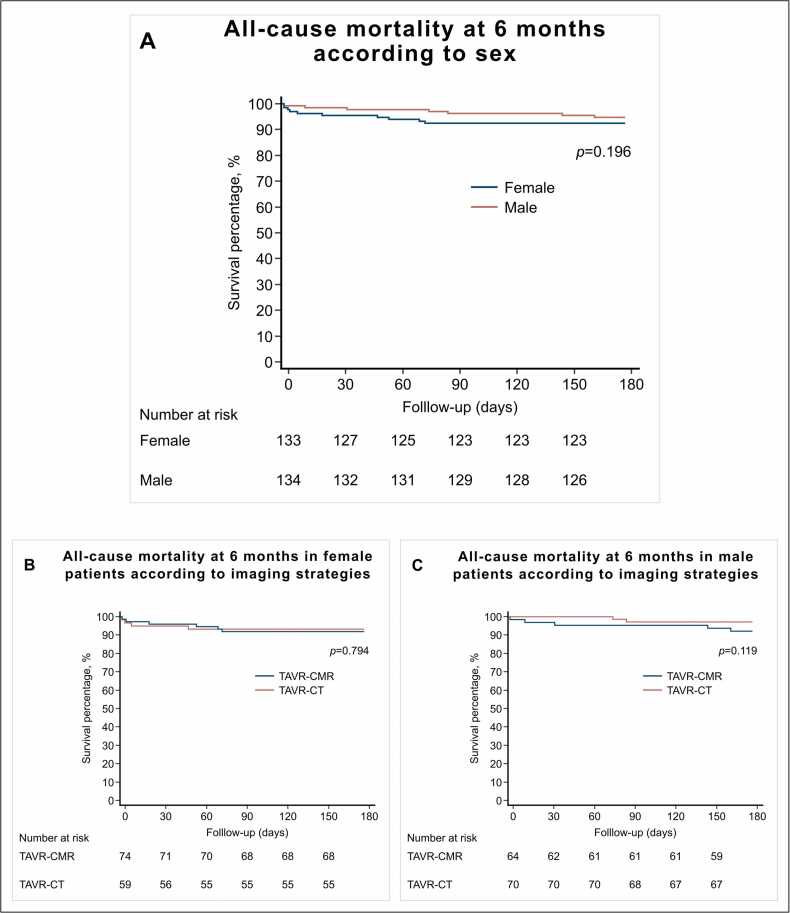
Cumulative incidence of all-cause mortality between sex and imaging strategy. *CMR* cardiovascular magnetic resonance*, CT* computed tomography*, TAVR* transcatheter aortic valve replacement. *p*-value for log-rank.

Secondary outcomes according to age groups and sex at discharge are shown in Table 7 and were comparable between groups. All-cause mortality at a median of 6 (IQR: 6–6) months did not differ between patients ≤82 years and patients >82 years (5.0% (7/139) vs. 11.0% (14/128), p = 0.074) (Fig. 3A), as well as in female and male patients (9.0% (12/133) vs. 6.7% (9/134), p = 0.484) (Fig. 4A).

Secondary outcomes according to age groups and imaging strategy at discharge are shown in Table 8 and were comparable between the CMR and CT groups. All-cause mortality at a median of 6 (IQR: 6–6) months did not differ between the CMR and CT groups in patients ≤82 years (5.3% (4/76) vs. 4.8% (3/63), p = 0.839) and in patients >82 years of age (12.9% (8/62) vs. 9.1% (6/66), p = 0.490) (Figs. 3B and 3C).

Secondary outcomes according to sex and imaging strategy at discharge are shown in Table 9 and were comparable between the CMR and CT groups. All-cause mortality at a median of 6 (IQR: 6–6) months did not differ between the CMR and CT groups in female patients (8.1% (6/74) vs. 10.2% (6/59), p = 0.680) and in male patients (9.4% (6/64) vs. 4.3% (3/70), p = 0.240) (Figs. 4B and 4C).

### 3.4. Sensitivity analyses using VARC-3 criteria

Sensitivity analyses using VARC-3 device success criteria demonstrated consistent findings for the primary outcome across both age groups, sex and their imaging strategies. In patients ≤82 years of age, the device success rate was 88.5% (123/138), compared to 85.2% (109/128) in patients >82 years of age (p = 0.420). When stratified by imaging strategy and age, device success rates were similar. In patients ≤82 years of age, the success rates were 89.5% (68/76) for CMR-guided TAVR and 87.3% (55/63) for CT-guided TAVR (p = 0.690). Similarly, in patients >82 years of age, device success was observed in 87.1% (54/62) of the CMR-guided group and 83.3% (55/66) of the CT-guided group (p = 0.550).

In female patients, the device success rate was 85.0% (113/133), compared to 88.8% (119/134) in male patients (p = 0.352). When stratified by imaging strategy and sex, device success rates were comparable. In female patients, the success rates were 87.8% (65/74) for CMR-guided TAVR and 81.4% (48/59) for CT-guided TAVR (p = 0.299). Similarly, in male patients, device success was observed in 89.1% (57/64) of the CMR-guided group and 88.6% (62/70) of the CT-guided group (p = 0.928).

## 4. Discussion

The TAVR-CMR trial was an investigator-initiated, prospective, randomized, open-label trial showing non-inferiority of CMR compared to CT in guiding TAVR procedures.

This secondary analysis of the TAVR-CMR trial showed that device implantation success according to VARC-2 criteria and short- to mid-term outcomes were comparable between CMR-guided and CT-guided TAVR across age groups and sexes. These data support the clinical value of CMR as an alternative imaging strategy for TAVR planning. This is important for older patients, who often have comorbidities that increase the risks associated with iodinated contrast exposure in CT-guided procedures [26], [27], [28], [29], [30]. At the same time, as TAVR expands to younger and lower-risk populations [2], the radiation-free nature of CMR offers a compelling advantage in minimizing the long-term risks associated with imaging.

Despite innate sex-related anatomical differences, no significant variations were observed in landing zone or access site parameters between imaging modalities for either sex. Moreover, device implantation success was consistent across both imaging strategies, regardless of sex. These findings highlight the efficacy and safety of CMR-guided TAVR in both male and female patients, further supporting its use as an alternative to CT-guided approaches.

### 4.1. Age-related aspects

Our findings provide important insights into the outcomes of different pre-procedural imaging strategies before TAVR in different age groups, an issue of growing importance as the procedure is increasingly considered for patients across a wider age spectrum [31]. The median age of our cohort was 82 years, which is consistent with other data from “real life” TAVR registries, such as the SwissTAVI and RISPEVA registries, where the median age ranges from 80 to 85 years [15], [32]. Older patients in our cohort (>82 years) had a significantly lower body-mass index and smaller aortic valve area compared to younger individuals, reflecting age-related anatomical changes observed in TAVR populations [15], [32], [33]. Despite these differences, no significant differences were found in device implantation success, all-cause mortality, stroke, or access-site complications between the age groups. These findings complement and extend previous observations, including those by Noguchi et al., which demonstrated that age alone is not a determinant of outcomes after TAVR when adjusted for factors such as frailty and chronic kidney disease [33].

The main finding of the current analysis is that when comparing the outcomes of CMR- and CT-guided TAVR, no significant differences were observed between the imaging strategies, irrespective of age. Therefore, these data provide important evidence across a range of patient ages to support CMR-guided TAVR planning as an alternative to CT-guided planning. CMR-guided TAVR planning may offer distinct advantages in specific populations. Older patients undergoing TAVR often have chronic kidney disease and the use of CMR eliminates the need for iodinated contrast agents, reducing the risk of contrast-induced acute kidney injury—a complication associated with poorer outcomes [29], [30]. This is particularly relevant as chronic kidney disease affects a substantial proportion of TAVR candidates, and AKI occurs in almost 50% of patients undergoing the procedure [26], [27], [28], [29], [30]. Given the relationship between contrast volume and kidney injury as well as subsequent poorer outcomes [34], [35], [36], minimizing the use of contrast during the TAVR “journey” is particularly important in these high-risk populations. It is therefore reassuring that TAVR planning using CMR is safe in terms of successful device implantation and all-cause mortality. Although incidences are very low, it must be emphasized that gadolinium-based contrast agents used for CMR can also cause potential clinical harm (e.g. nephrogenic systemic fibrosis, gadolinium deposition). In addition, as TAVR expands into younger and lower-risk populations, minimizing long-term risks associated with radiation exposure becomes important. Unlike CT, CMR is radiation-free, addressing concerns about cumulative radiation dose that are particularly relevant in younger patients with longer life expectancies [12], [16].

While the cohort consisted mainly of octogenarians, limiting generalizability to younger patients, current guidelines recommend TAVR over surgical aortic valve replacement in patients aged ≥75 years [11]. In this context, patients aged 75–80 years represent a relevant “younger” TAVR population and accounted for 30% of the study cohort. Although younger patients may benefit most from a radiation-free imaging approach, TAVR remains predominantly performed in older populations [11], [32]. In these patients, with a high pre-test probability of coronary artery disease, invasive coronary angiography is currently the recommended method of coronary artery disease assessment prior to TAVR [11]. However, CT performed as part of periprocedural planning may offer advantages over CMR by providing additional coronary information, particularly as emerging evidence suggests that photon-counting CT enables high-resolution coronary assessment even in patients with severe coronary calcification or prior stent implantation and could potentially replace the need for invasive coronary angiography in the future [37].

Beyond its safety profile, CMR has demonstrated comparable accuracy to CT for key procedural parameters, such as aortic annulus sizing and access route evaluation [12], [14], [25]. This study provides novel evidence from randomized patients supporting CMR as a feasible alternative imaging modality regardless of patient age. CT offers some advantages in visualizing calcifications, a critical factor in TAVR planning that influences platform selection and deployment strategies [38]. This is particularly relevant in older patients, who often exhibit a higher burden of aortic valve calcification due to age-related degenerative changes [12], [15]. Although CMR lacks the ability to assess calcifications directly, our findings demonstrated no significant differences in procedural success or clinical outcomes between the two imaging strategies, even in the very elderly patient group. This suggests that the difference in calcification assessment between the two methods, although important, does not negate the clinical utility of CMR in procedural planning.

### 4.2. Sex-related aspects

In TAVR trials, female patients represent a higher proportion of enrolled patients than in other cardiovascular studies with up to 53% in meta-analyses [39], [40], [41], and this is reflected in our data. Females undergoing TAVR were shown to present with a lower incidence of cardiovascular interventions, stroke, and peripheral vascular disease history [41]. This is in line with our findings, reporting lower percentages of previous cardiovascular interventions in female patients. However, female TAVR patients were reported to have worse renal function [42], a trend also seen in our cohort, which may be particularly relevant for CT-planning using iodinated contrast [43].

Beyond clinical conditions, female patients inherently have smaller landing zone and access site parameters [18], [19], [20], [21]. In particular, the aortic annular size as one of the most important parameters for TAVR planning is smaller in female patients [18], [44]. Furthermore, female patients show smaller iliac and femoral artery diameters [18]. These anatomical differences can lead to different rates of procedural complications. In fact, oversizing in small annuli can lead to annulus rupture, a life-threatening procedural complication of TAVR [45], [46]. Small artery diameters are associated with increased risk of access site complications, which were reported to occur more frequently in female patients [40], [41]. Concerning the relevant sex differences in aortic valve and vascular anatomy, it is crucial to evaluate the potential impact of sex on imaging assessment while establishing a new TAVR-guiding strategy. Recently, the TAVR-CMR trial demonstrated the non-inferiority of CMR compared to CT in guiding TAVR [47]. The present secondary analysis specifically assessed the relevance of sex in this regard and found no differences in landing zone parameters such as annulus area, diameter, and perimeter between CT- and CMR-guided TAVR planning in both sexes. Furthermore, in terms of minimum diameters of peripheral vessels, we found no differences between CT- or CMR-guiding in female patients. In male patients, minimum diameters of left and right femoral arteries were comparable, while iliac arteries were larger in the CMR-guiding arm. Although statistically significant, these absolute differences were small and had no clinical implications as the diameters were relatively large (median measurements between 8 and 10 mm) and therefore did not affect the trans-femoral feasibility of final device implantation.

The primary endpoint of TAVR-CMR, device implantation success according to the VARC-2 criteria, was also comparable between the CT- and CMR-guidance in the present sex-specific secondary analysis. The primary composite and all singular endpoints, including the absence of procedural mortality, correct valve positioning, or intended device performance, were comparable between the imaging strategies regardless of sex. Thus, it confirms very high implantation success rates and good clinical outcomes immediately after TAVR consistently in both guidance strategies irrespective of sex. In addition, a sensitivity analysis of the primary endpoint was performed using the updated VARC-3 criteria that include additional components compared to the VARC-2 criteria such as freedom from surgery or intervention related to the device or to a major vascular or access-related or cardiac structural complication [23]. In this further analysis, we found consistent results for the primary outcome. Furthermore, key secondary outcomes at discharge showed no differences between CT- and CMR-guided imaging in both sexes. Altogether, these findings confirm the efficiency and safety of CMR-guidance for high-device implantation success in both female and male patients and suggests the implementation of CMR-guidance for TAVR planning as equivalent alternative to the conventional CT-guided approach.

## 5. Limitations

Although our study provides valuable insights into the age-related outcomes of CMR-guided versus CT-guided TAVR from the first and only randomized trial in this field, several limitations must be acknowledged. The secondary nature of our analysis may limit the generalizability of our findings. In addition, stratification of participants by age and imaging strategy reduces subgroup sizes, which limits statistical power. Deeper insights into the age spectrum by splitting the cohort into more than two age groups were hampered by the moderate overall sample size. Future research should focus on prospective, multicenter studies with larger and more diverse populations to further validate our findings and explore the long-term outcomes associated with CMR-guided TAVR. The median age of 82 years reflects current clinical practice in Europe, and consequently very few patients in our analysis were younger than 75 years. Therefore, our results cannot be generalized to age groups <75 years. Specific studies in this patient population would be desirable, as the indication for TAVR in this age group is growing. Although invasive coronary angiography is recommended for the assessment of coronary artery disease before TAVR, CT might have advantages over CMR in providing additional information on coronary status. Since the CMR protocol for the TAVR-CMR trial did not include cine sequences, data on CMR-derived left and right ventricular volumes and function were not available.

## 6. Conclusion

CMR-guided TAVR provided comparable outcomes to CT-guided TAVR in terms of device implantation success and all-cause mortality regardless of patient age or sexes.

These observations provide additional evidence to support the use of CMR for TAVR planning in both elderly patients with comorbidities and younger patients where minimizing long-term risks becomes more relevant. Furthermore, these results confirm that both imaging strategies are equally effective and safe for male and female patients.

## Funding

The study was supported by grants from the “Research Grant from the Society for the Advancement of Cardiovascular Research in Tyrol,” “10.13039/501100015797Austrian Society of Cardiology“ and the “10.13039/501100009968Tiroler Wissenschaftsförderung”. This research was funded in part by the 10.13039/501100002428Austrian Science Fund (FWF) 10.55776/KLI772. For open access purposes, the author has applied a CC BY public copyright license to any author accepted manuscript version arising from this submission.

## Author contributions

**Bernhard Metzler**: Writing – review & editing, Visualization, Validation, Supervision, Project administration, Funding acquisition, Data curation, Conceptualization. **Ivan Lechner**: Writing – review & editing, Writing – original draft, Methodology, Investigation, Funding acquisition, Formal analysis, Data curation, Conceptualization. **Martin Reindl**: Writing – review & editing, Writing – original draft, Visualization, Validation, Methodology, Investigation, Funding acquisition, Formal analysis, Data curation, Conceptualization. **Axel Bauer**: Writing – review & editing, Supervision, Resources. **Magdalena Holzknecht**: Writing – review & editing, Formal analysis, Data curation. **Alex Kaser**: Writing – review & editing, Project administration, Data curation. **Fritz Oberhollenzer**: Writing – review & editing, Writing – original draft, Methodology, Investigation, Formal analysis, Data curation, Conceptualization. **Sebastian J. Reinstadler**: Writing – review & editing, Validation, Supervision, Project administration, Methodology, Data curation, Conceptualization. **Christina Tiller**: Writing – review & editing, Methodology, Formal analysis, Data curation. **Gert Klug**: Writing – review & editing, Resources, Data curation, Conceptualization. **Agnes Mayr**: Writing – review & editing, Visualization, Validation, Resources, Methodology, Formal analysis, Data curation, Conceptualization. **Ronald K. Binder**: Writing – review & editing, Validation, Resources. **Can Gollmann-Tepeköylü**: Writing – review & editing, Data curation, Conceptualization.

## Ethics approval and consent

Ethical approval for this study was granted by the local research ethics committee (Ethics Committee of the Medical University of Innsbruck, Austria; AN3775 281/4.15 405/5.2; 4480a). Written informed consent was given by all patients before study inclusion.

## Consent for publication

Not applicable.

## Declaration of Competing Interest

The authors declare that they have no known competing financial interests or personal relationships that could have appeared to influence the work reported in this paper.

## Data Availability

The data analyzed in this study can be obtained from the corresponding author with a reasonable request.
