# Supplementary material for: Age and sex-related outcomes in cardiovascular magnetic resonance versus computed tomography-guided transcatheter aortic valve replacement: a secondary analysis of a randomized clinical trial
Source: J Cardiovasc Magn Reson. 2025 Mar 13;27(1):101882. doi: 10.1016/j.jocmr.2025.101882 (PMC12138550; doi:10.1016/j.jocmr.2025.101882)
Supplement: Supplementary file 1 — Supplementary material [file mmc1.docx]

**SUPPLEMENTAL MATERIAL**

**SUPPLEMENTAL TABLES**

**Table S1:** **Sub-Analysis of Anatomical and Hemodynamic Parameters of the Aortic Valve**

|  | Total population  (n=267) | TAVR-CMR group  (n=138) | TAVR-CT group  (n=129) | p-value |
| --- | --- | --- | --- | --- |
| **Anatomical parameters** | |  |  |  |
| Moderate or severe calcification of aortic valve leaflets and left ventricular outflow tract | 241 (90) | 123 (92) | 118 (92) | 0.906 |
| Moderate or severe calcification of the right access site | 107 (41) | 51 (39) | 56 (45) | 0.316 |
| Moderate or severe calcification of the left access site | 120 (45) | 56 (43) | 64 (51) | 0.176 |
| **Hemodynamic parameters** | |  |  |  |
| Aortic valve area, cm² | 0.6 (0.5-0.8) | 0.6 (0.5–0.8) | 0.6 (0.5–0.8) | 0.848 |
| Mean gradient, mmHg | 40 (30-46) | 40 (29–47) | 40 (30–46) | 0.982 |
| Moderate or severe aortic regurgitation | 63 (24) | 35 (25) | 28 (22) | 0.482 |
| Left ventricular ejection fraction, % | 57 (49-64) | 55 (48–63) | 58 (51–64) | 0.104 |
| Stroke volume index, mL/m² | 30 (25-39) | 30 (24–37) | 31 (25–40) | 0.265 |

TAVR=transcatheter aortic valve replacement, CMR=Cardiac magnetic resonance, CT=Computed tomography.
